# Practical Points in Nursing

**Published:** 1903-07

**Authors:** 


R. and L.

---

Practical Points in Nursing. For Nurses in Private Practice. With an Appendix containing Rules for Feeding the Sick; Recipes for Invalid Food and Beverages; Weights and Measures; Dose List; and a full Glossary of Medical Terms and Nursing Treatment. By Emily A. M. Stoney, late Superintendent of the Training School for Nurses, Carney Hospital, South Boston, Mass. Third edition, thoroughly revised. Handsome 12mo of 458 pages, fully illustrated, including 8 colored and half-tone plates. Philadelphia, New York, London: W. B. Saunders & Company. 1903. Cloth, $1.75 net.

The publishers lay especial stress upon the fact that in the preparation of the present edition this work has been completely revised from beginning to end. For example, the sections treating on infectious diseases, the treatment of the common poisonings, etc., have been entirely recast and rewritten.

By the aid of this well-written and well-arranged little work any nurse of average intelligence should be able to instruct herself as to the best means of meeting the various emergencies likely to occur in medical and surgical cases while awaiting the arrival of the doctor.

R. and L.

